# Engineered Red Blood Cell‐Derived Extracellular Vesicles With Klotho Peptide Protect the Kidney From Fibrosis

**DOI:** 10.1002/jex2.70159

**Published:** 2026-07-03

**Authors:** Tunahan Ergunay, Alessia Brossa, Michela Arena, Stefania Bruno, Alessandro Gori, Anne Metje van Genderen, Rosalinde Masereeuw, Benedetta Bussolati

**Affiliations:** ^1^ Department of Medical Sciences University of Turin Turin Italy; ^2^ Department of Molecular Biotechnology and Health Sciences University of Turin Turin Italy; ^3^ Istituto di Scienze e Technologie Chimiche “GiulioNatta” (SCITEC) Consiglio Nazionale delle Ricerche Milan Italy; ^4^ Utrecht Institute for Pharmaceutical Sciences, Division of Pharmacology Utrecht University Utrecht The Netherlands

**Keywords:** chronic kidney disease, exosome therapy, kidney fibrosis, organ‐on‐chip, TGFβ, therapeutic peptide

## Abstract

Chronic kidney disease is characterized by progressive tubular injury and fibrosis, ultimately leading to irreversible loss of renal function. Extracellular vesicles (EVs) have emerged as promising therapeutic platforms due to their biocompatibility and engineering potential. In this study, we engineered red blood cell–derived EVs to deliver an active peptide (RBC‐EVKP1) from the anti‐aging hormone klotho, as a targeted antifibrotic strategy. EVs were isolated from healthy donors and surface‐functionalized with the peptide. Fibrosis was evaluated in proximal tubular epithelial cells exposed to TGFβ and in a 3D proximal tubule‐on‐chip model. RBC‐EVKP1 treatment significantly attenuated TGFβ‐induced fibrotic and mesenchymal gene expression, reduced extracellular matrix accumulation, and suppressed SMAD signaling. In addition, RBC‐EVKP1 reduced cell migration and preserved epithelial organization. In the tubule‐on‐chip system, engineered EVs maintained cytoskeletal integrity and reduced injury‐associated marker expression under fibrotic conditions. Collectively, these findings demonstrate that klotho‐engineered EVs effectively inhibit TGFβ‐driven fibrosis and preserve tubular integrity, highlighting a promising therapeutic strategy for targeting kidney fibrosis.

## Graphical abstract

In this study, we engineered red blood cell–derived extracellular vesicles with a Klotho‐derived peptide (RBC‐EVKP1) to target kidney fibrosis. Engineered RBC‐EVKP1, through inhibition of TGF‐β2/TGFβ receptor binding, and downstream signaling, reduced fibrotic and mesenchymal marker expression and extracellular matrix accumulation in proximal tubular epithelial cells while preserving tubular integrity. These findings highlight RBC‐EVKP1 as a promising therapeutic strategy against kidney fibrosis.

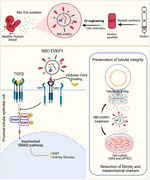

## 1 Introduction

Chronic kidney disease (CKD) is a major global health burden, affecting approximately 10% of the adult population and representing a leading cause of morbidity and mortality worldwide (Francis et al. 2024). CKD is characterized by a progressive and irreversible decline in renal function, which frequently culminates in end‐stage kidney disease, requiring renal replacement therapy. Despite diverse etiologies, CKD progression converges on common pathological mechanisms, among which renal fibrosis plays a central role. Fibrosis disrupts renal architecture, compromises tubular integrity, and ultimately drives functional deterioration, yet effective antifibrotic therapies remain unavailable.

Renal fibrosis is orchestrated by a complex interplay between tubular epithelial cells, fibroblasts, inflammatory mediators, and extracellular matrix remodeling (Li et al. 2022). Among profibrotic pathways, transforming growth factor‐β (TGFβ) signaling is a key driver of epithelial–mesenchymal transition (EMT), myofibroblast activation, and excessive extracellular matrix (ECM) deposition (Sureshbabu et al. 2016). Persistent activation of the TGFβ/SMAD axis promotes irreversible tissue scarring, making this pathway an attractive but challenging therapeutic target. To date, although fibrosis has been estimated to contribute to nearly 45% of all deaths worldwide (Wynn 2008), direct inhibition of TGFβ signaling has been limited by toxicity (Mitra et al. 2025) highlighting the need for targeted and tissue‐specific approaches.

Extracellular vesicles (EVs) are lipid bilayer‐enclosed nanoparticles secreted by almost all eukaryotic cells and found in various biological fluids (EL Andaloussi et al. 2013). EVs have emerged as promising tools for translational medicine due to their intrinsic biocompatibility, stability, and capacity to deliver bioactive molecules in a cell‐free manner (Yang et al. 2024). Importantly, EVs can be engineered to display therapeutic cargos on their surface, enabling targeted modulation of receptor‐mediated signaling pathways. Red blood cell (RBC)‐derived EVs represent a particularly attractive platform because of their scalability, safety profile, and lack of nuclear or mitochondrial DNA (Usman et al. 2018).

Klotho is an anti‐aging and renoprotective protein whose expression is markedly reduced during kidney injury and CKD progression (Buchanan et al. 2020). Indeed, the kidney is responsible for releasing the majority of soluble klotho circulating in plasma and urine (Buchanan et al. 2020). Recent evidence demonstrates that a peptide derived from the extracellular KL1 domain of klotho can protect against renal fibrosis by inhibiting TGFβ signaling through receptor binding (Yuan et al. 2022). Building on these findings, we engineered RBC‐EVs with a klotho‐derived peptide to selectively interfere with TGFβ signaling and explored the antifibrotic potential of engineered RBC‐EVs using complementary in vitro and organ‐on‐chip models.

## 2 Materials and Methods

### 2.1 RBC‐EV Isolation

RBC‐EVs were isolated from fresh blood samples of healthy donors provided by the Blood Bank of Città della Salute e della Scienza di Torino. All samples were obtained after informed consent, in accordance with the Declaration of Helsinki, and approval by the internal review board of the Blood Bank. RBC‐EVs were obtained from approximately 50 mL whole blood of a single donor, as previously described (Usman et al. 2018). Briefly, Ficoll density gradient (Sigma Aldrich) centrifugation was used to isolate RBCs, which were diluted 1:1 in PBS (Euroclone) with 0.1 g/l CaCl_2_, and finally treated with 10 µM calcium ionophore (Sigma Aldrich) overnight at 37°C. RBCs and cell debris were then removed by sequential centrifugation at 600 g for 20 min, 1.600 g for 15 min, 2.700 g for 15 min, and 10.000 g for 30 min at 4°C. Obtained supernatants were passed through 0.45 µm filtration units (Nalgene), and subjected to ultracentrifugation using a fixed angle rotor (TY70Ti, Beckman Coulter) at 50.000 g for 2 h at 4°C. RBC‐EVs were resuspended in 1% DMSO in PBS and stored at −80°C for further analyses.

### 2.2 Klotho Peptide Synthesis

The klotho peptide was synthesized by standard Fmoc solid‐phase peptide synthesis and designed for EV engineering applications. To this end, a short PEG spacer was introduced, and an N‐terminal cysteine residue was included to enable maleimide‐based conjugation (see the following section). Full details of peptide synthesis and purification are reported in the Supplementary Information.

### 2.3 RBC‐EV Engineering

To link the klotho peptide to RBC‐EV membrane, 1 × 10^11^ RBC‐EVs were incubated with 10 mg SMCC (Sigma‐Aldrich) for 30 min at RT in 500 µL PBS at pH 7.2. A total of 170 mg of klotho peptide were then added and samples were incubated for 60 min at RT in 1 mL PBS at pH 7.6. RBC‐EVKP1 was then purified with an ultracentrifugation step at 50.000 g for 2 h at 4°C. After ultracentrifugation, engineered EVs were resuspended in RPMI 1% DMSO and stored at −80°C for further analyses. Engineered EVs are then characterized in terms of size and concentration using Nanosight (NS300, Malvern Panalytical).

### 2.4 Cell Culture

Human proximal tubular epithelial cells (HK2) were cultured in DMEM Low Glucose medium (EuroClone) supplemented with 10% fetal bovine serum, 20 mM L‐glutamine, and 1% penicillin/streptomycin. Cells were maintained in a humidified incubator at 37°C with 5% CO_2_. Human conditionally immortalized proximal tubular epithelial cells (ciPTECs; Cell4Pharma, Oss, the Netherlands) were cultured in DMEM/F12 medium without phenol red (Gibco), supplemented with ITS (insulin 5 µg/mL, transferrin 5 µg/mL, sodium selenite 5 ng/mL; I1884, Sigma‐Aldrich), 36 ng/mL hydrocortisone (H6909, Sigma‐Aldrich), 10 ng/mL epidermal growth factor (EGF; 9644, Sigma‐Aldrich), and 40 pg/mL 3‐iodothyronine (T5516, Sigma‐Aldrich). Cells were initially expanded at 33°C and, following expansion, were incubated at 37°C for 1 week to allow full maturation, as described previously (Wilmer et al. 2010).

### 2.5 TGFβ and EV Treatments

After 24 h of cell seeding with a density of 40.000 cell/cm^2^, HK2 cells were pretreated with serum‐free medium to enhance EV uptake. The following day, cells were treated with 10 ng/ml human recombinant TGFβ1 (hereafter referred to TGFβ; T7039, Sigma‐Aldrich) and RBC‐EVKP1 at a dose of 50.000 EV/cell for 24 h in complete medium, unless otherwise indicated. The EV dose used in our experiments was selected based on preliminary dose–response studies evaluating the modulation of fibrosis‐related markers in tubular epithelial cells. A range of 10.000 to 100.000 EVs/cell was tested based on previously reported dosages used in EV‐mediated renal injury and fibrosis models (Franzin et al. 2022). Among the tested concentrations, 50.000 EVs/cell in HK2 cells and 40.000 EVs/cell in ciPTECs provided the most consistent antifibrotic effects while maintaining optimal cell viability and experimental reproducibility. To control potential effects of EV content, a group treated with unmodified RBC‐EVs (RBC‐EVCTL) was included. Human ciPTECs were seeded at a density of 55.000 cell/cm^2^ and treatments were initiated after 1 week of maturation. Cells were exposed to 10 ng/ml TGFβ and RBC‐EVKP1 at 40.000 EV/cell for 1 week, with four doses administered by refreshing the complete medium throughout the treatment period. To reduce donor‐specific variability, at least three different EV donor sources were included among biological replicates.

### 2.6 RNA Isolation and Real‐Time PCR

Total RNA was isolated from HK2 and ciPTECs using TRIzol reagent (Ambion) according to the manufacturer's instructions. Complementary DNA (cDNA) was synthesized from the isolated RNA using MultiScribe Reverse Transcriptase (Thermo Fisher Scientific). Gene expression analysis was performed by real‐time PCR in a 20 µL reaction containing 5 ng of cDNA, specific oligonucleotide primers, and Power SYBR Green PCR Master Mix (Applied Biosystems). GAPDH was used as a housekeeping gene, and relative fold changes in gene expression were calculated for all samples using the ΔΔCt method. For RNA isolation from cells cultured on hollow‐fiber membrane, cells were first trypsinized and collected by centrifugation. The resulting cell pellet was resuspended in 300 µL of TRIzol reagent, and the subsequent RNA extraction and cDNA synthesis procedures were carried out as described above.

### 2.7 Cytofluorimetric Analysis

To assess successful EV engineering, RBC‐EVs were linked to an APC klotho peptide. A total of 5 × 10^9^ RBC‐EVKP1^APC^ were then diluted in 100 µL PBS (Euroclone) previously filtered with 0.1 µm membrane pore filters (Merck Millipore), and stained for 15 min with ISO‐PE or CD9‐PE antibodies (Miltenyi Biotec). Samples were further diluted with 200 µL PBS, and immediately acquired using the Cytek Aminis CellStream Cytometer. The percentage of KP1‐APC/CD9‐PE positive events was calculated. Flow cytometry was performed on ciPTECs detached with enzyme‐free cell dissociation solution (S‐014‐C, Merck). Cells were pelleted, washed with HBSS, and then resuspended in 0.1% BSA in HBSS. Cells were incubated for 15 min with the following antibodies and their respective isotype controls: anti‐CD324‐APC (130‐099‐723, Miltenyi Biotec) and anti‐CD325‐PE (350805, BioLegend). After incubation, cells were centrifuged and resuspended in blocking buffer. Stained cells were analyzed using a FACScelesta flow cytometer, and data were processed with FACSDiva software (BD Biosciences).

### 2.8 Immunofluorescence

To assess RBC‐EVKP1 uptake, HK2 cells with a density of 30.000 cell/cm^2^ were plated on coverslips. The following day, cells were treated with TGFβ and 100.000 EV/cell RBC‐EVKP1^APC^. After 24 h, cells were washed three times with PBS, fixed with 4% paraformaldehyde for 15 min at 37°C, and washed again three times with PBS. Cells were then permeabilized with 0.1% Triton X‐100 in PBS for 30 min at room temperature, followed by blocking in 1% BSA with 0.05% Triton X‐100 in PBS for 1 h at room temperature. Phalloidin‐FITC (1:1000, P5282, Sigma Aldrich) in blocking buffer was applied for 1 h to stain actin filaments. After washing three times with PBS, nuclei were stained with DAPI. Finally, cells were washed, mounted with mounting medium, sealed with nail polish, and stored at ‐20°C until imaging. RBC‐EVKP1 localization was visualized by confocal microscopy (SP5, Leica) using LAS‐AF software. A similar procedure was applied to mature ciPTECs, in which the PBS was replaced with HBSS.

To visualize ECM components, HK2 cells (50.000 cell/cm^2^) were plated on coverslips in serum‐free medium and treated with TGFβ and RBC‐EVKP1 during seeding. After blocking, cells were incubated with primary antibodies against αSMA (A5228, Sigma Aldrich) and fibronectin (ab2413, Abcam) in blocking buffer. Following three washes with PBS, cells were incubated with Alexa Fluor 488 (A11029, Invitrogen) and Alexa Fluor 594 (A21442, Invitrogen) secondary antibodies, washed again, nuclei were stained with DAPI (10236276001, Sigma Aldrich), and mounted with a mounting medium. For ciPTEC‐OAT1 cultured on HFM, fibers were removed from the chip and placed on a chambered slide (μ‐slide, Ibidi) in small segments. Immunofluorescence staining was performed using the same protocol as for 2D cultures. At the end, fibers were mounted in mounting medium.

### 2.9 Transwell Migration and Wound‐Healing Assays

To assess cell migration, HK2 cells were resuspended in serum‐free medium and seeded onto 0.4 µm Transwell inserts (353495, Corning) placed in a multiwell plate containing complete medium in the lower chamber. Cells were simultaneously treated with TGFβ and RBC‐EVKP1. After 24 h, the medium was discarded, and non‐migrated cells on the upper surface of the membrane were removed using a cotton swab. Migrated cells were fixed with methanol for 10 min, air‐dried, and stained with 0.2% crystal violet in ethanol for 20 min. Following staining, membranes were washed with water, allowed to dry, and visualized using light microscopy. For the wound‐healing assay, HK2 cells were seeded in multiwell plates and serum‐starved. A straight scratch was created using a pipette tip, and detached cells were removed by washing with PBS. Complete medium was then added, and cells were treated with TGFβ and RBC‐EVKP1. Wound closure was monitored under a brightfield microscope (Axio observer Z1, Zeiss) for up to 48 h. The wound closure rate was quantified by measuring the wound area using ImageJ software.

### 2.10 SBE Reporter Assay

TGFβ/SMAD pathway activity was assessed using the SBE reporter assay (60654, BPS Bioscience) following the manufacturer's instructions. Briefly, 50.000 cell/cm^2^ HEK293T cells were plated on black, clear‐bottom 96‐well microplates and cultured until they reached approximately 90% confluency. Cells were then transfected with the 4X‐SBE luciferase vector using Lipofectamine reagent and incubated for 24 h. The following day, the medium was replaced with 0.5% FBS‐containing medium, and cells were treated with TGFβ and RBC‐EVKP1. After 24 h of treatment, luciferase activity was measured using a D‐luciferin (122796, Perkin Elmer) and quantified with a plate reader (GloMax, Promega).

### 2.11 Proximal Tubule‐on‐Chip Culture

The proximal tubule‐on‐chip (PToC) system was based on a previously, step‐wise established platform (Jansen et al. 2015, Jochems et al. 2020, Giordano et al. 2025). MicroPES‐type TF10 hollow capillary membranes were cut, placed into a chip system, and sterilized in 70% ethanol for 45 min under UV light. Membranes were then washed three times with HBSS and coated with L‐3,4‐dihydroxyphenylalanine (L‐DOPA, 2 mg/mL in 10 mM Tris buffer, pH 8.5, D9628, Merck) by incubation at 37°C for 5 h. After being washed with HBSS, hollow fiber membranes (HFMs) were incubated with human collagen IV solution (25 µg/mL in HBSS, C5533, Merck) for 1 h at 37°C. Fibers were then washed three times with HBSS to get rid of unbound collagen. Human ciPTECs overexpressing the organic anion transporter 1 (OAT1) (Nieskens et al. 2018) were seeded onto the coated HFMs at a density of 1×10^6^ cells/ml in chips. After 3 h, unattached cells were removed by refreshing the medium. Cells were cultured at 33°C for 2–3 days to form a continuous monolayer on the HFMs and then transferred to 37°C for 7–14 days to obtain fully matured artificial kidney tubules. To visualize the formation of a continuous tubular structure, cells were labeled with a membrane dye (Vybrant DiO, Invitrogen) following the manufacturer's instructions. Briefly, 5 µL DiO reagent was mixed with 1 mL of a 1×10^6^ cells/ml suspension and incubated for 20 min at 37°C. After incubation, cells were centrifuged and washed with warm medium to remove excess dye. Labeled cells were then seeded onto HFMs, and monolayer formation was subsequently monitored using fluorescence microscopy (THUNDER Imager, Leica).

### 2.12 Data Analysis

Results are generally expressed as mean ±SEM, as indicated. Statistical analysis was performed using ANOVA followed by Dunnett's multiple comparison test or by Student's t test when required. A *p* value <0.05 was considered significant.

## 3 Results

### 3.1 Engineering RBC‐Derived EVs With Klotho Peptide

**FIGURE 1:**
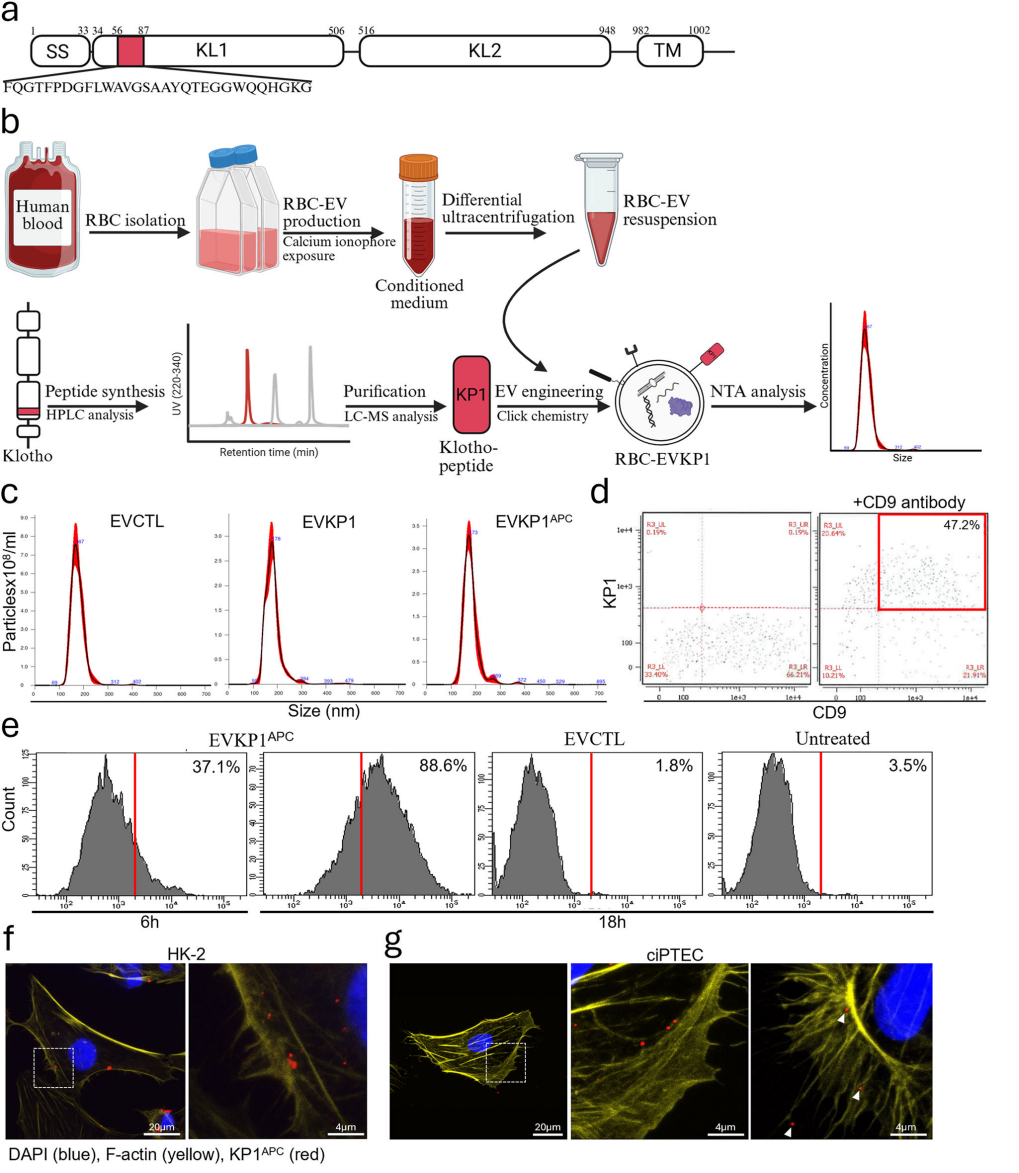
Engineering of RBC‐EVs with KP1 peptide and their uptake by human kidney cells. (a) Structure of the full‐length klotho protein. The protein comprises four domains: A signal sequence (SS), KL1, KL2 and a transmembrane domain (TM). The peptide and its sequence used in this study are indicated with a red marker. (b) Schematic illustration of RBC‐EV isolation and surface engineering with the KP1 peptide. The image is created in Biorender. (c) Nanoparticle tracking analysis of unmodified and engineered RBC‐EVs. (d) Flow cytometric assay indicating the CD9+ RBC‐EV population with the KP1 peptide. The corresponding quadrant is highlighted by the red rectangle (e) Flow cytometric assay showing changes in the KP1+ ciPTEC population after 6 and 18 h. IF images of RBC‐EVKP1APC‐treated HK2 cells (f) and ciPTECs (g) highlighted their localization on the cell membrane. Triangles on the last image represent EVs localized on cell membrane protrusions.

A 30 amino acid peptide, corresponding to a sequence within the KL1 klotho domain, was first synthesized (Figure 1a). In parallel, EVs were isolated from RBCs obtained from healthy donors through overnight exposure to calcium ionophore and subsequent differential ultracentrifugation (Figure 1b). Cytofluorimetric single EV analysis showed the expression of CD9 and CD47 by RBC‐EVs (Figure S1). Subsequently, RBC‐EVs were functionalized with the klotho peptide via maleimide chemistry, allowing covalent conjugation of the peptide to the EV surface. A short PEG spacer was incorporated into the peptide construct to ensure adequate spatial exposure of the klotho peptide on the EV surface. The engineered vesicles were designated as RBC‐EVKP1. Nanoparticle tracking analysis showed that RBC‐EVs size (around 167 nm) did not significantly vary after KP1 engineering (Figure 1c). To verify successful functionalization, a fluorophore (APC)‐labeled KP1 peptide was conjugated to the EVs, and flow cytometry analysis confirmed the efficient attachment of KP1 to CD9^+^ RBC‐EVs (Figure 1d).

To examine cellular uptake, RBC‐EVs were incubated with human ciPTEC, a conditionally immortalized tubular cell line able to grow at 33°C and fully differentiate at 37°C. Flow cytometry analysis revealed that 37% of cells internalized RBC‐EVKP1 after 6 h, increasing to 88% after 18 h (Figure 1e). Consistent with these results, immunofluorescence imaging demonstrated efficient uptake of RBC‐EVKP1 by both HK2 (Figure 1f) and ciPTEC (Figure 1g), showing multiple KP1 signals distributed at the plasma membrane and within the cytoplasm. Of note, we also observed RBC‐EV accumulation near cell surface protrusions, possibly filopodia, which may facilitate vesicle trafficking and internalization.

### 3.2 RBC‐EVKP1 Prevents Kidney Fibrosis and Epithelial‐Mesenchymal Transition

**FIGURE 2:**
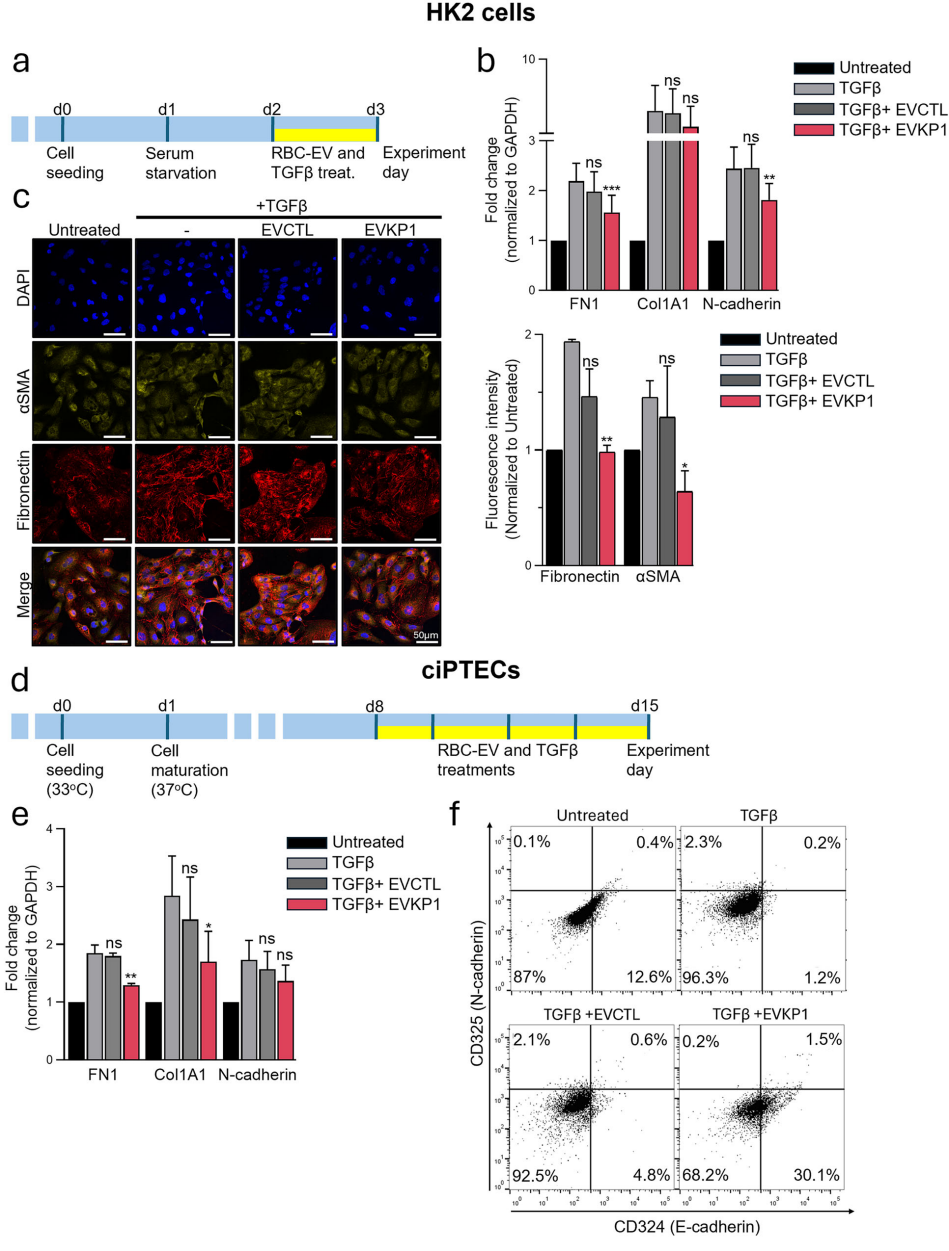
Fibrosis and EMT induction by TGF are prevented by RBC‐EVKP1 treatment in HK2 cells and ciPTECs. (a) HK2 RBC‐EV and TGF treatment experimental design. The treatment duration is indicated by a yellow line. (b) Real‐time PCR analysis of fibrosis (fibronectin and col1a1) and mesenchymal (n‐cadherin) genes. (c) Immunofluorescence imaging of fibrosis markers αSMA (yellow) and fibronectin (red), with nuclear counterstaining using DAPI (blue). For quantification, fluorescence intensity is calculated and normalized to untreated. The data represent the normalized value of the mean fluorescence intensity to untreated group, analysis made from at least 3 independent biological replicates and error bars represent SEM. Two‐way ANOVA followed by Dunnett's multiple comparisons test was used to compare experimental conditions with the TGFβ treatment (**P < 0.01, *P < 0.05, ns, not significant). (d) Experimental design for RBC‐EV and TGFβ treatment for ciPTECs. Treatments  indicated in the lanes were given to matured cells as four doses. (e) Real‐time PCR analysis of fibrosis (fibronectin and col1a1) and mesenchymal (n‐cadherin) genes. (f) Flow cytometry analysis of epithelial (CD324) and mesenchymal (CD325) proteins. (b, e) Fold changes represent ΔΔCt values that were normalized to GAPDH and untreated. Data are represented as mean with SEM from at least 3 independent biological replicates. Statistical analysis represents differences compared to TGFβ treatment and performed with Student's t test with paired values. (***P < 0.001, **P < 0.01, *P < 0.05, ns, not significant).

The antifibrotic potential of engineered RBC‐EVKP1 was evaluated by treating HK2 cells with TGFβ in the presence or absence of RBC‐EVKP1 (Figure 2a). Real‐time PCR analysis showed that TGFβ stimulation markedly upregulated the expression of fibrotic and mesenchymal markers, whereas co‐treatment with RBC‐EVKP1 significantly reduced their expression (Figure 2b). Consistently, immunofluorescence analysis revealed increased deposition of fibrosis‐associated ECM components, including fibronectin and αSMA, following TGFβ exposure, while RBC‐EVKP1 co‐treatment effectively prevented their expression (Figure 2c).

To validate these findings, the experiments were extended to conditionally immortalized ciPTECs, which grow at 33°C and differentiate at 37°C, as they more closely resemble human proximal tubular epithelial cells than HK2 cells and retain renal physiological features (Jenkinson et al. 2012, Klatt et al. 2025). As we observed fibrosis induction after one week of TGFβ treatment following cellular maturation (data not shown), the experimental setup was adjusted accordingly (Figure 2d). ciPTECs were exposed to TGFβ with or without RBC‐EVKP1 for one week, receiving four doses throughout the treatment period. Real‐time PCR analysis confirmed results consistent with those obtained in HK2 cells, showing strong induction of fibrotic and mesenchymal markers by TGFβ, that was efficiently counteracted by RBC‐EVKP1 co‐treatment (Figure 2e). Moreover, flow cytometry analysis demonstrated that RBC‐EVKP1 treatment decreased the mesenchymal marker n‐cadherin and restored the epithelial marker e‐cadherin (Figure 2f). Collectively, these findings indicate that RBC‐EVKP1 effectively mitigates TGFβ‐induced fibrotic responses and preserves epithelial characteristics in proximal tubular epithelial cells.

### 3.3 Functional Outcome of RBC‐EVKP1 on Kidney Fibrosis

**FIGURE 3:**
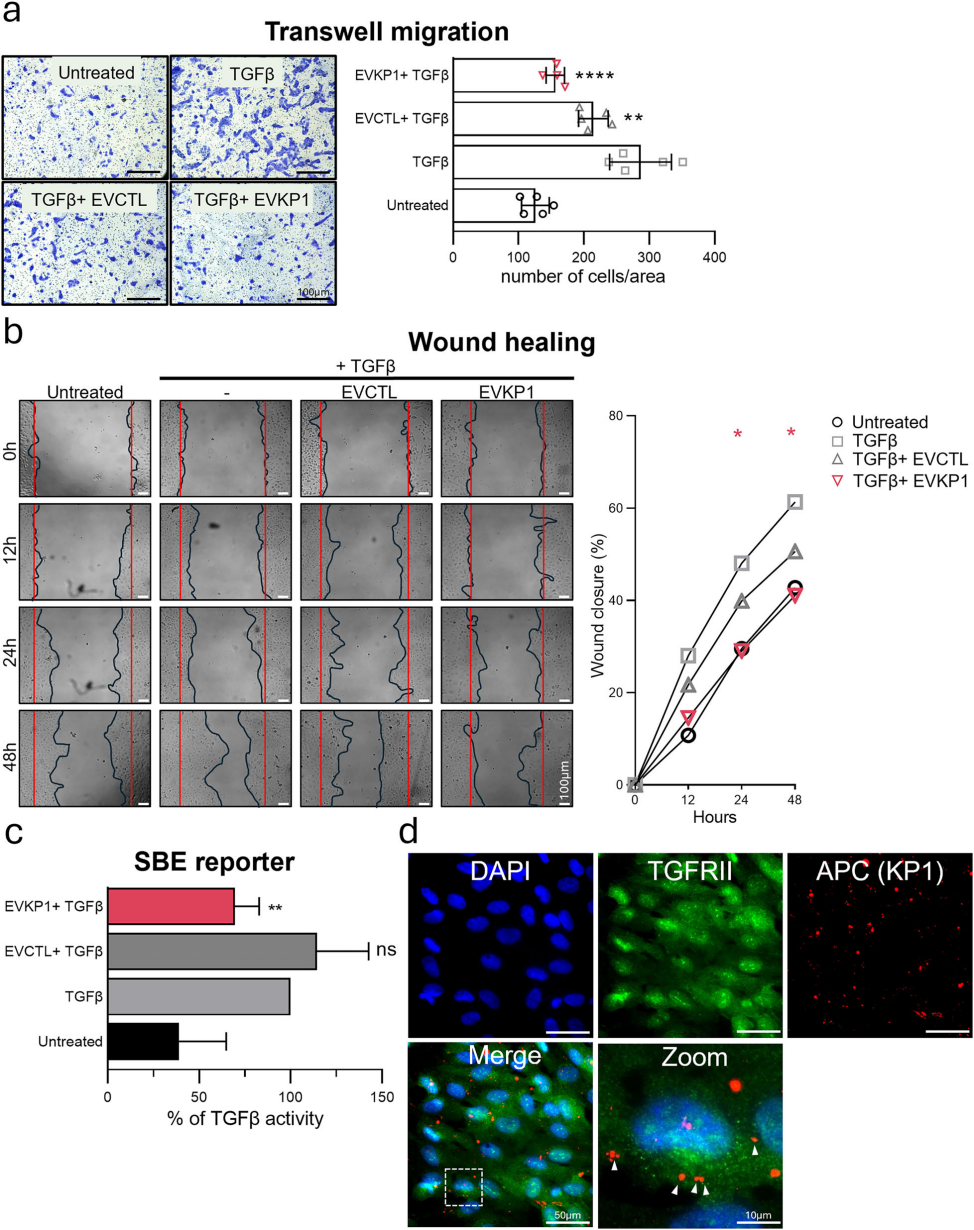
RBC‐EVKP1 inhibits TGFβ‐driven profibrotic signaling and mesenchymal characteristics. (a) Transwell migration assay. Images represent migrated cells on the lower part of the cell culture insert after cell fixation and staining with crystal violet. Data represents number of cells in an area. (b) Wound‐healing assay. Wound closure was monitored for up to 48 h, and the rate of closure was calculated based on the change in wound area over time. The red line indicates the initial wound boundary, and the black markings illustrate progressive wound closure. (c) SBE reporter assay to test TGFβ pathway activity in HEK‐293T cells. The luminescence signal of TGFβ treatment is considered as 100% activitiy. (a, b, c) Each data consist of at least three biological replicates, error bars represent SEM. Analysis represents one‐way ANOVA for panel a and two‐way ANOVA for panel b with multiple comparisons and paired t‐tests for panel c. Significance indicators denote comparisons relative to the TGFβ‐treated group. (****P < 0.0001, **P < 0.01, *P < 0.05, ns, not significant). (d) Immunofluorescence assay showing co‐localization of TGFRII and KP1. It is indicated with an arrowhead.

We next examined the functional effects of RBC‐EVKP1 on the mesenchymal behavior of HK2 cells. Transwell migration assays revealed a marked increase in the number of migrating cells upon TGFβ stimulation (Figure 3a). Treatment with unmodified RBC‐EVs partially reduced this migratory response, whereas co‐treatment with RBC‐EVKP1 almost completely prevented the TGFβ‐induced increase in cell migration. Consistently, wound‐healing assays showed that TGFβ‐treated HK2 cells exhibited accelerated wound closure, with approximately 50% closure at 24 h and 60% at 48 h (Figure 3b). In contrast, RBC‐EVKP1 co‐treatment significantly inhibited this effect, limiting wound closure to about 40% after 48 h. These findings indicate that RBC‐EVKP1 effectively suppresses the acquisition of mesenchymal traits and the enhanced motility induced by TGFβ in HK2 cells. A modest reduction in migration was also observed in cells treated with unmodified RBC‐EVs, likely reflecting the intrinsic bioactive potential of RBC‐derived vesicles.

To further investigate the underlying mechanism, TGFβ/SMAD signaling activity was assessed using a SMAD‐binding element (SBE) reporter assay. Due to low transfection efficiency in HK2 and ciPTECs, the assay was performed in HEK‐293T cells according to the manufacturer's recommendations. TGFβ stimulation led to strong activation of the pathway, which was significantly suppressed by RBC‐EVKP1 co‐treatment (Figure 3c). This observation suggests that RBC‐EVKP1 interferes with TGFβ/SMAD signaling, potentially through klotho peptide interaction with the TGFβ receptor, thereby preventing ligand binding, as previously reported (Buchanan et al. 2020). Supporting this mechanism, immunofluorescence analysis demonstrated colocalization of APC‐labeled RBC‐EVKP1 with TGFRII (Figure 3d).

### 3.4 RBC‐EVKP1 Protects Tubular Integrity

**FIGURE 4:**
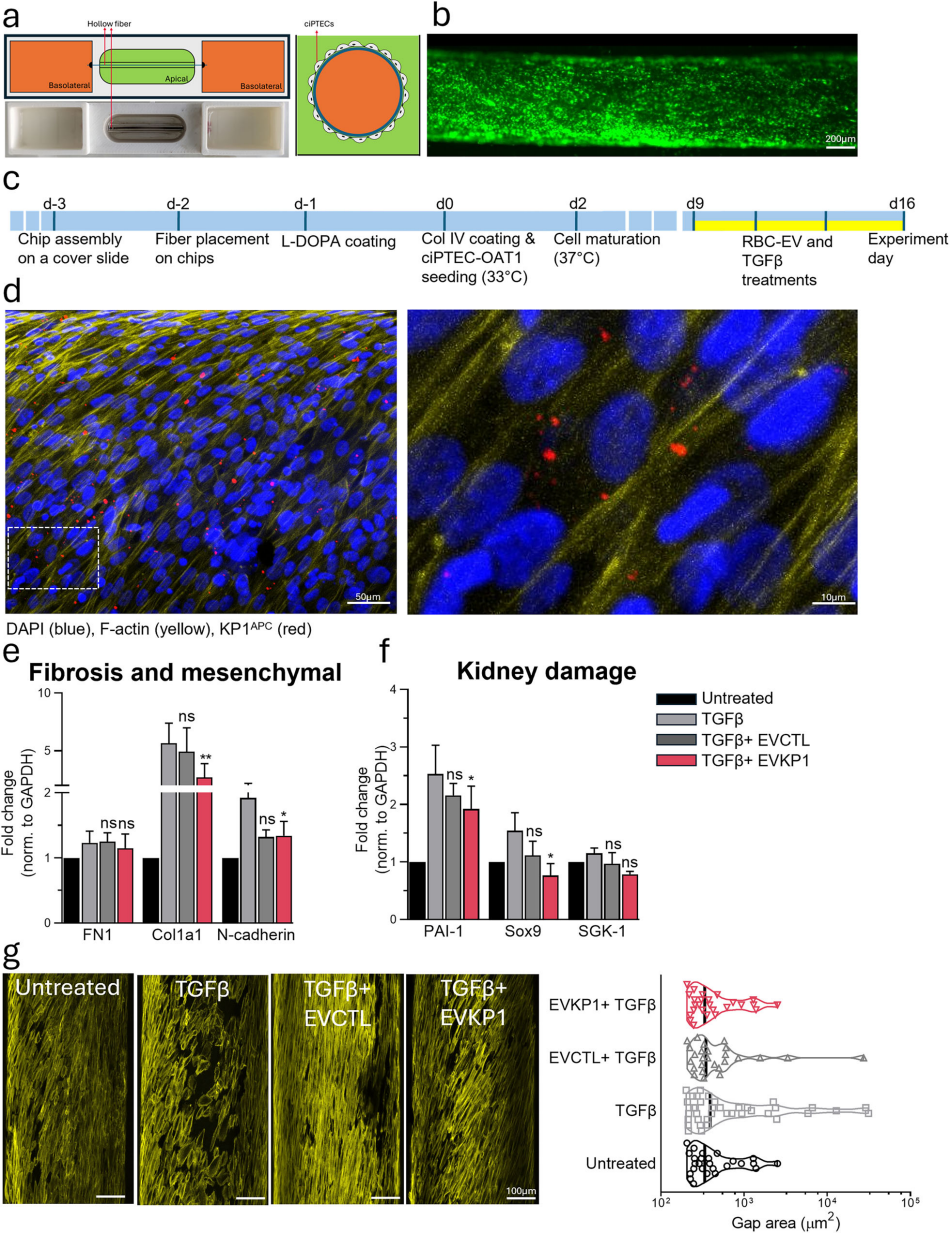
Human proximal tubule‐on‐chip (PToC) system is damaged by TGFβ‐induced kidney fibrosis and is restored by RBC‐EVKP1 treatment. (a) Chip design (Jochems et al. 2020). Chambers on each side allow the medium to flow along the basolateral side of the fiber (orange), and a central chamber containing the fiber and apical perfusate (green). ciPTEC‐OAT1 cells cover the outside of the fiber. (b) DiO staining shows that the fiber is fully covered by cells at the end of cell maturation (c) Experimental timeline of ciPTEC cells on the PToC system. TGFβ and EV treatments are started at day 9 and maintained for 7 days with 4 doses. (d) Immunofluorescence image showing a ciPTEC monolayer around the fiber and cellular uptake of APC‐labeled RBC‐EVKP1. The area indicated by dashed lines is shown in a zoomed view. Real‐time PCR analysis of (e) fibrosis (fibronectin and col1a1), mesenchymal (n‐cadherin) and (f) kidney damage‐related genes. (e, f) Fold changes represent ΔΔCt values are normalized to the value of GAPDH and the untreated. Data are represented as mean with SEM from at least 3 independent biological replicates. Statistical analysis represents differences compared to TGFβ treatment, and Student's t test is performed with paired values (**P < 0.01, *P < 0.05, ns, not significant). (g) Immunofluorescence assay on hollow fiber showing actin filaments (yellow) through the fiber. Presence of black areas and cells in different directions indicates disrupted tubular integrity. The violin plot represents the distribution of gap areas along the fibers, with each data point corresponding to an individual gap. The median value is indicated by a black horizontal line.

Given the known regenerative properties of klotho and the antifibrotic effects demonstrated in previous experiments, we next investigated whether RBC‐EVKP1 could preserve proximal tubular integrity under fibrotic conditions. To this end, we employed a 3D chip system, using the proximal tubular cell line ciPTEC‐OAT1 seeded onto HFM (Figure 4a). This apical‐out microfluidic platform enables basolateral perfusion and regulated molecular transfer toward the apical compartment via epithelial cell–mediated transport. Similar to 2D ciPTEC culture, after establishing a confluent monolayer at 33°C, the chips were transferred to 37°C to promote cellular maturation. In this phase, cells create a continuous monolayer around the tubule (Figure 4b). Subsequently, mature ciPTEC‐OAT1 were treated with TGFβ to induce fibrosis and co‐treated with RBC‐EVKP1 as in the in vitro assays (Figure 4c).

Immunofluorescence analysis confirmed continuous monolayer formation along the hollow fiber and verified efficient uptake of APC‐labeled RBC‐EVKP1 by ciPTEC‐OAT1 (Figure 4d). Real‐time PCR analysis revealed that RBC‐EVKP1 treatment reduced the expression of fibrotic and mesenchymal markers compared to cells treated with TGFβ alone (Figure 4e). In addition, the expression of tubular injury markers, PAI‐1, SOX9, and SGK1, was markedly increased following TGFβ treatment but was attenuated in the presence of RBC‐EVKP1 (Figure 4f).

To assess structural integrity within the tubular constructs, phalloidin staining was performed to visualize the organization of the actin cytoskeleton. Control tubules displayed continuous, well‐aligned actin filaments and uniform epithelial coverage along the fiber (Figure 4g). In contrast, TGFβ‐treated tubules exhibited pronounced cytoskeletal disruption, characterized by multiple large gaps along the fiber suggestive of cell detachment or loss, impaired uniform cell directionality, and the acquisition of a mesenchymal cell morphology. Notably, co‐treatment with RBC‐EVKP1 preserved the structural architecture of the tubules by effectively preventing TGFβ‐induced cytoskeletal and cellular disorganization and maintaining tubular integrity with markedly fewer gaps. Although treatment with unmodified RBC‐EVs also contributed to partial preservation of tubular integrity, large gaps remained evident along the fiber. To further assess tubular integrity, we performed an albumin leakage assay; however, no significant increase in protein leakage was detected in TGFβ‐treated tubules, indicating that the structural disturbances occurred without measurable barrier impairment under TGFβ‐induced fibrotic conditions.

## 4 Discussion

In this study, we demonstrate that RBC–derived EVs engineered with a klotho‐derived peptide effectively counteract TGFβ‐driven fibrotic responses and preserve tubular epithelial integrity in human kidney models.

Renal fibrosis represents the final common pathological outcome of CKD irrespective of the initiating insult, and sustained activation of the TGFβ/SMAD signaling cascade is a key driver of this process. The antifibrotic and renoprotective potential of klotho has been previously demonstrated in animal models of CKD (Li et al. 2025, Li et al. 2024), and we have shown that klotho engineered EVs displayed therapeutic effects in a murine model of acute kidney injury (Grange et al. 2020). Mechanistically, klotho was also reported to competitively bind to the TGFβ type II receptor, thereby inhibiting activation of the TGFβ/SMAD signaling pathway (Buchanan et al. 2020). Although klotho has been recognized as an attractive therapeutic target, its short half‐life and lack of specific targeting limit clinical application.

Here, we exploited a mechanistically defined antifibrotic klotho‐derived peptide, KP1, that selectively interferes with TGFβ receptor signaling (Yuan et al. 2022), and delivered it through a biocompatible EV‐based platform to enhance antifibrotic efficacy. Notably, this peptide has been shown to suppress the expression of senescence‐associated genes (Zhang et al. 2024), supporting its broader renoprotective potential. Beyond KP1 peptide activity *per se*, EV‐based delivery offers distinct therapeutic advantages. The presentation of bioactive ligands at high density on the EV surface may facilitate efficient receptor engagement and clustering, resulting in stronger and more sustained signaling modulation compared with free peptide administration (Mohammadi et al. 2023). In particular, the EV membrane provides a nanoscale platform that may confine receptors at the EV‐cell contact site, and promote a dense local concentration of receptors, which can amplify receptor‐level effects (Jahnke and Staufer 2024). These properties make EVs an attractive and versatile delivery system for enhancing the stability, targeting, and biological efficacy of klotho‐based antifibrotic therapies.

A strength of this study lies in the use of RBC–derived EVs as a delivery vehicle. RBC‐EVs are naturally produced and can be easily obtained from healthy donors through repeated collections (Ma et al. 2023). For therapeutic applications, their production can also be artificially induced to yield high quantities (Biagiotti et al. 2024). RBC‐EVs offer several advantages for translational applications, including scalability, intrinsic biocompatibility, and the absence of nuclear and mitochondrial DNA, reducing safety concerns related to horizontal gene transfer (Chiangjong et al. 2021). Supporting their therapeutic potential, a recent study engineered RBC‐EVs with siRNAs targeting acute kidney injury and demonstrated effective delivery to injured proximal tubules via a Kim‐1 binding peptide, resulting in reduced inflammation and fibrosis in mouse models of acute kidney injury (Tang et al. 2021).

The results of the present study, performed in different human settings, indicate the ability of RBC‐EVKP1 to suppress SMAD activation and EMT, highlighting the potential of TGF‐beta receptor‐level modulation. Unmodified RBC‐EVs showed minimal and non‐significant effects on fibrosis markers and functional outcomes, supporting the concept that the antifibrotic activity observed is rather driven by the conjugated klotho peptide than by the native cargo of RBC‐EVs.

Beyond conventional 2D cell culture systems, we employed a human PToC model to assess the impact of RBC‐EVKP1 on tubular architecture under fibrotic stress. This microphysiological platform recapitulates key structural features of renal tubules and enables the evaluation of epithelial integrity in a dynamic and spatially organized context (Faria et al. 2023). Under physiological conditions, tubular epithelial cells form a continuous unidirectional monolayer with actin filaments aligned in parallel, reflecting structural polarity and cohesion. However, following TGFβ stimulation, cells lost their epithelial morphology, exhibited altered shapes, and in some cases detached from the fiber or underwent cell death. Despite these changes, protein leakage could not be detected. These findings may reflect an early stage of tubular injury, during which RBC‐EVKP1 treatment preserves cytoskeletal organization, and mitigates early structural alterations. The observed effects could be of relevance from a translational perspective, where measurable functional decline often manifests at later stages of pathology, following substantial structural damage (Quinn et al. 2021).

Despite these promising findings, several limitations should be acknowledged. The experiments were conducted using in vitro and organ‐on‐chip systems, whereas the biodistribution and therapeutic efficacy of engineered EVs should also be validated in vivo or in more complex models. Of interest, previous studies have demonstrated the preferential localization of RBC‐derived EVs in the kidneys of mouse models, supporting their potential for renal targeting (Tang et al. 2021, Pat et al. 2022). In this study, the free form of the KP1 peptide was not included due to the limited stability and short half‐life of small peptides, as well as their susceptibility to proteolytic degradation. In contrast, EVs can enhance the stability of proteins and peptides and protect them from degradation, while enhancing receptor engagement and clustering, making them promising carriers for therapeutic delivery. Nevertheless, direct comparison of the antifibrotic efficacy of free KP1 and EV‐conjugated KP1 would provide valuable insight and should be addressed in future studies. In addition, the use of human samples introduces inherent inter‐batch variability. For translation toward clinical applications, EV production workflows must be standardized, and the feasibility of autologous EV‐based treatments should be evaluated in light of donor‐dependent differences in EV characteristics and biological activity.

## 5 Conclusion

In summary, we successfully engineered RBC‐derived extracellular vesicles with the KP1 peptide and demonstrated that they inhibit TGFβ‐induced signaling, reduce fibrotic and mesenchymal changes, and preserve tubular epithelial integrity in proximal tubular cells. Mechanistically, these effects highlight the ability of KP1‐engineered RBC‐EVs to directly interfere with the TGFβ pathway and prevent progression of fibrotic remodeling. From a translational perspective, the combination of RBC‐EVs’ biocompatibility, stability, and scalable production with the targeted activity of the KP1 peptide offers a promising therapeutic platform for the treatment of kidney fibrosis.

## Author Contributions

**Tunahan Ergunay**: conceptualization, investigation, writing – original draft, methodology, writing – review and editing, formal analysis. **Alessia Brossa**: investigation, formal analysis, methodology. **Michela Arena**: investigation, methodology. **Stefania Bruno**: investigation, methodology. **Alessandro Gori**: investigation, methodology. **Anne Metje van Genderen**: investigation, methodology, formal analysis. **Rosalinde Masereeuw**: conceptualization, supervision, writing – review and editing, funding acquisition. **Benedetta Bussolati**: conceptualization, investigation, funding acquisition, writing – review and editing, supervision.

## Funding

This research was funded by PNRR MUR – M4C2 – CN3 SPOKE 8 Invest. 1.4 MUR CN00000041 CUP D13C22001310001 and Ministero dell'Istruzione, dell'Università e della Ricerca.

## Ethics Statement

Blood samples of healthy donors provided by the Blood Bank of Città della Salute e della Scienza di Torino, were obtained after informed consent in accordance with the Declaration of Helsinki, and approval by the internal Blood Bank review board.

## Conflicts of Interest

The authors declare no conflict of interest with the contents of this article. Rosalinde Maseru is co‐inventor of the cell line ciPTEC‐OAT1, for which the patent is held by Radboud University Nijmegen, the Netherlands.

## Supporting information




**Supporting Information**: jex270159‐sup‐0001‐SuppMat.docx

## Data Availability

The data in this study are available in the article itself, the supplementary materials or upon request from the corresponding author upon reasonable request.
