# Supplementary material for: Engineered Red Blood Cell‐Derived Extracellular Vesicles With Klotho Peptide Protect the Kidney From Fibrosis
Source: J Extracell Biol. 2026 Jul 3;5(7):e70159. doi: 10.1002/jex2.70159 (PMC13329256; doi:10.1002/jex2.70159)
Supplement: Supplementary file 1 — Supporting Information: jex270159‐sup‐0001‐SuppMat.docx [file JEX2-5-e70159-s001.docx]

**Engineered Red Blood Cell-Derived Extracellular Vesicles with Klotho Peptide Protect the Kidney from Fibrosis**

Tunahan Ergunay^1^, Alessia Brossa^2^, Michela Arena^1^, Stefania Bruno^1^, Alessandro Gori^3^, Anne Metje van Genderen^4^, Rosalinde Masereeuw^4^, Benedetta Bussolati^1,*^

## Supplementary Material and Methods

## Peptide synthesis

Peptides were assembled by stepwise microwave-assisted Fmoc-SPPS on a Biotage ALSTRA Initiator+ peptide synthesizer, operating in a 0.12 mmol scale on a Rink amide resin (C-terminus amidated peptides). Resins were swelled prior to use with a NMP/DCM mixture (1:3) for 1 hour. Activation and coupling of Fmoc-protected amino acids was performed using Oxyma 0.5M / DIC 0.5M (1:1:1), with a 4 equivalent excess over the initial resin loading. Coupling steps were performed for 10 minutes at 60°C. Deprotection steps were performed by treatment with a 20% piperidine solution in DMF at room temperature for 5min. Following each coupling or deprotection step, peptidyl-resins were washed with DMF (4x). Peptides were cleaved from the resin using a TFA 90%, water 5%, thioanisole 2.5%, TIS 2.5% mixture (2 hours, RT), and then precipitated in cold diethyl ether. Crude peptides were collected by centrifugation and washed with further cold diethyl ether to remove scavengers. Linear precursors were HPLC analyzed and purified as described below.

## RP-HPLC analysis and purification

Analytical and semi-preparative reversed phase high performance liquid chromatography (RP-HPLC) were carried out on a Shimadzu Prominence HPLC system equipped with a multichannel detector. A Phenomenex Jupiter 5µ C18 90Å column (150 × 4.6 mm) was used for analytical runs and a Phenomenex Jupiter 10µ C18 90Å (250 × 21.2 mm) for peptide purification. Data were recorded and processed with LabSolutions software. 5-100 % linear gradient eluent B at a flow rate of 0.5 mL/min was used for analytic purposes (20 min run). eluent A = H_2_O/ 3 % CH_3_CN / 0.07 % TFA, eluent B = 70 % CH_3_CN/ 30 % H_2_O/ 0.07 % TFA. UV detection was recorded in the 220-340 nm range. Peptide purification was performed by preparative RP-HPLC at a flow rate of 14 mL/min using a 100% A🡪 30% B linear gradient over 40 min. Pure fractions (>95%) were combined and lyophilized. LC-MS analysis was then performed on pure fractions (Table S1).

**Supplementary Table 1**

| **Peptide** | **Sequence** | **MS calc.** | **MS found** |
| --- | --- | --- | --- |
| KP1 | C-(O_2_Oc)-FQGTFPDGFLWAVGSAAYQTEGGWQQHGKG | MW: 3476.6  [M+2]^2+^=1739.3; [M+3]^3+^=1159.9  [M+4]^4+^=870.1 | [M+3]^3+^=1159.8  [M+4]^4+^=870.1 |

**Table S1**. Molecular mass of synthetic peptides as determined by mass spectrometry analysis (ESI-MS). Standard amino acids are represented by conventional one letter code. O2Oc: 8-amino-3,6-dioxaoctanoic acid.

**Supplementary Figure 1**


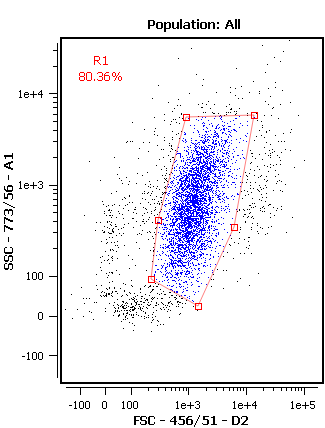

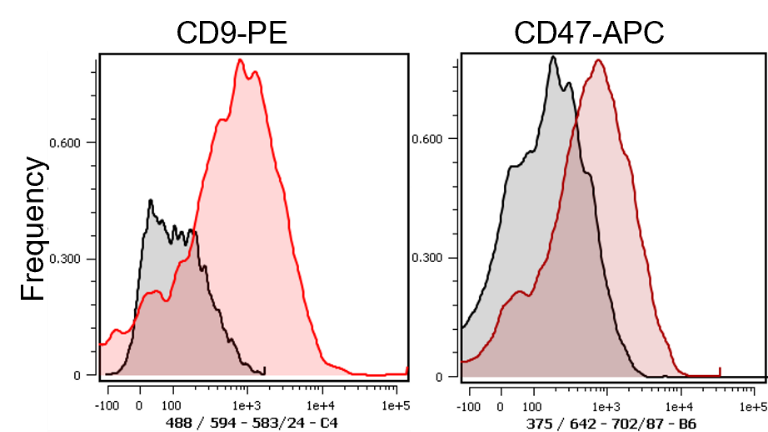


**Fig. S1**. Flow cytometric characterization of RBC-EVs. The EV population was gated based on forward- and side-scatter physical parameters (left panel). The representative histograms show the surface marker expression on RBC-EVs. The red histograms indicate CD9-PE–positive (middle panel) and CD47-APC–positive (right panel) RBC-EVs, while the grey histograms represent the corresponding isotype controls.
